# Strain and Electric Field Controllable Schottky Barriers and Contact Types in Graphene-MoTe_2_ van der Waals Heterostructure

**DOI:** 10.1186/s11671-020-03409-7

**Published:** 2020-09-21

**Authors:** Yu Lan, Li-Xin Xia, Tao Huang, Weiping Xu, Gui-Fang Huang, Wangyu Hu, Wei-Qing Huang

**Affiliations:** 1 College of Physics and Electronic Engineering, Hengyang Normal University, Hengyang, 421002 China; 2 Department of Physics, Kashi University, Kashi, 844006 China; 3 Department of Applied Physics, School of Physics and Electronics, Hunan University, Changsha, 410082 China; 4 Dingcheng District Power Supply Branch of Changde Power Supply Company, State Grid, Changde, 415100 China; 5 School of Materials Science and Engineering, Hunan University, Changsha, 410082 China

**Keywords:** Schottky barrier, Graphene-MoTe_2_ heterostructure, External electric field, Strain, First-principles calculations

## Abstract

Two-dimensional (2D) transition metal dichalcogenides with intrinsically passivated surfaces are promising candidates for ultrathin optoelectronic devices that their performance is strongly affected by the contact with the metallic electrodes. Herein, first-principle calculations are used to construct and investigate the electronic and interfacial properties of 2D MoTe_2_ in contact with a graphene electrode by taking full advantage of them. The obtained results reveal that the electronic properties of graphene and MoTe_2_ layers are well preserved in heterostructures due to the weak van der Waals interlayer interaction, and the Fermi level moves toward the conduction band minimum of MoTe_2_ layer thus forming an *n* type Schottky contact at the interface. More interestingly, the Schottky barrier height and contact types in the graphene-MoTe_2_ heterostructure can be effectively tuned by biaxial strain and external electric field, which can transform the heterostructure from an *n* type Schottky contact to a *p* type one or to Ohmic contact. This work provides a deeper insight look for tuning the contact types and effective strategies to design high performance MoTe_2_-based Schottky electronic nanodevices.

## Introduction

Two-dimensional (2D) layered crystals have attracted increasing interest due to their novel physical properties and potential applications in various fields since the discovery of graphene [1]. Unconventional features and performance, such as half-integer quantum Hall effect [2], Klein tunneling [3], and superconductivity [4], have been discovered in various 2D materials. For graphene, however, the Dirac cone type band structure without a band gap near the Fermi level hinders its direct applications in transistors. This has stimulated the searching for alternative materials from other 2D materials [5–14] with versatile properties, among which layered transition metal dichalcogenides (TMDs) have gained extensive attention. The band gaps of TMDs can be tuned from about 0.8 eV to 2.0 eV and are comparable with that of conventional semiconductors, enabling TMDs especially good candidates for optoelectronic applications. Being similar to graphite, most of TMDs are layered-structure materials with van der Waals (vdW) interaction between layers, thus can be exfoliated to few layers or a single layer [15, 16]. It is found that TMDs have thickness-dependent characteristics and would undergo an indirect-direct band gap transition [16, 17] when they are changed from bulk to few layers or monolayer. Monolayer TMDs have several structures, such as H phases and T phases (or T′ phases), while the H phases usually exhibit semiconducting characteristics.

As a member of TMDs, bulk MoTe_2_ includes three interesting phases: hexagonal (2H, semiconducting) phase [18], monoclinic (1 T′, metallic) phase [19], and octahedral (T_d_, type II Weyl semimetal) phase [20, 21], in which 2H-phase is the most stable one. 2H-phase MoTe_2_ has an indirect band gap of about 1.0 eV for bulk and a direct band gap of about 1.1 eV for monolayer [22, 23], which indicates that the band gap is almost independent of the number of layers and it can be applied for the near-infrared photodetectors. For convenience, in the following text, 2H-MoTe_2_ is simply referred as MoTe_2_. Compared with other TMDs, MoTe_2_ has many advantages, for example, the conductivity is lower [24], Seebeck coefficient is higher [24], and the sensing abilities are better [18, 25]. Combining the advantages of MoTe_2_ and graphene, fabricating a type of heterostructure by graphene and MoTe_2_ for device applications could be considered. Actually, recently vertical heterostructures based on 2D-layered-structure materials have been attracted increasing interests [26–33] due to the absence of dangling bonds at the surfaces of isolated components and weak Femi level pinning. For graphene-TMDs-based vertical heterostructures, experiments have confirmed their excellent high on-off ratio, high photo-response, low dark current, and good quantum efficiency [34–38], as compared with simple TMDs-based types. Though most of the reported graphene-TMDs-based vertical heterostructures are constructed with other TMDs, such as MoS_2_, some experiments have investigated the graphene-MoTe_2_ heterostructure [39–43] due to the unique electronic and optical properties of MoTe_2_. It was reported [39] that the on-off ratio of the graphene-MoTe_2_ vertical heterostructure is as high as ~(0.5 − 1) × 10^−5^, and the photo responsivity can reach 20 mAW^−1^, which are comparable to the corresponding values of graphene-MoS_2_ device. Later, based on graphene-MoTe_2_-graphene vertical vdW heterostructure, a near-infrared photodetector was fabricated [40, 42] with a superior performance, including high photoresponsivity, high external quantum efficiency, rapid response and recovery processes, and free from an external source−drain power supply compared to other layered semiconductor photodetectors. Then, a graphene-MoTe_2_ vdW vertical transistor which exhibits suitable V-shaped ambipolar characteristics [41] was reported. Hence, the graphene-MoTe_2_ heterostructures are promising candidates for optoelectronic nanodevices with high responsivity, high-speed, and flexible. In this sense, it is essential to carry out a theoretical investigation on graphene-MoTe_2_ vertical heterostructure which has not been reported yet.

For the metal-semiconductor heterostructure, the contact type (Schottky contact or Ohmic contact) has to be considered, because it determines the existence of rectifying characteristics or not for the heterostructure. For the Schottky contact, the Schottky barrier height (SBH) would play a key role on the behaviors of the corresponding devices [44] and has been investigated intensely. In order to achieve high performance for actual device applications, it would be desirable that SBH can be tuned. Many strategies have been proposed to modulate the SBH, among which applying an external electric field and biaxial strain are the most common ways.

In this paper, based on first-principles calculations, electronic structure, and external electronic field and strain dependence of the SBH of graphene-MoTe_2_ heterostructure have been investigated. The calculated results demonstrate that the electronic properties of graphene and MoTe_2_ monolayer are preserved quite well after being vertically stacked up as a heterostructure. The Schottky barrier of the heterostructure can be changed between *p* type and *n* type by applying an external electric field or strain, and the heterostructure can even reach the Ohmic contact when the external electric field or strain is strong enough.

## Computational Methods

First-principle calculations have been carried out using the Vienna Ab-initio Simulation Package (VASP) [45–47] based on density functional theory (DFT). The projector augmented wave (PAW) [48] pseudopotentials were applied to model ion-electron interaction and the Perdew-Burke-Ernzerhof (PBE) generalized gradient approximation (GGA) [49] was used to treat electron exchange correlation. For all calculation, the DFT-D2 [50] method of Grimme representing the vdW interaction term are employed and the plane-wave cutoff energy is set to 600 eV. The convergence threshold is set to 10^−6^ eV for energy and 0.01 eV/Å for force. The Brillouin zone *k*-point mesh is set as 9 × 9 × 1 within the Monkhost-Pack scheme. A vacuum space of 25 Å along the *z* direction is adopted to avoid the interaction between the neighboring layers. As it was revealed that the spin-orbit coupling effect on band structures of 2H-MoTe_2_ is very weak [51], all of the calculations do not consider the spin-orbit coupling.

**Fig. 1:**
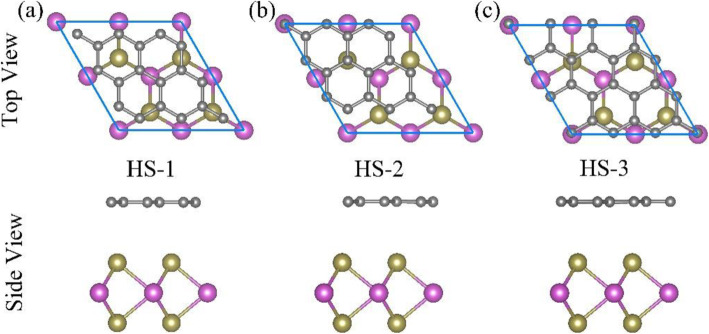
Top view and side view of three typical stacking modes for the graphene-MoTe_2_ heterostructure: (**a**) HS-1, (**b**) HS-2, (**c**) HS-3. The gray, pink, and green balls represent the carbon, molybdenum, and tellurium atoms, respectively

The graphene-MoTe_2_ heterostructure is constructed by graphene and MoTe_2_ monolayer via stacking the two 2D materials along the vertical direction. Both graphene and MoTe_2_ adopt the hexagonal lattice and their lattice parameters are 2.46 Å [52] and 3.52 Å [53], respectively. Hence, the lattice mismatch is lower than the previous criterion of 5%. According to the structures of graphene and MoTe_2_ monolayer, here, three typical stacking modes are considered: HS-1, HS-2, and HS-3, which are shown in Fig. 1. For HS-1 stacking mode, one Te atom just locates under the hollow site of the graphene lattice; for HS-2, one Te atom sits under one C atom site of the graphene lattice; for HS-3, one Te atom sits under another nonequivalent C atom site of the graphene lattice.

When the strain dependence of the SBH is investigated, strain is applied equally along the zigzag and armchair directions of graphene, respectively.

## Results and Discussion

**Fig. 2:**
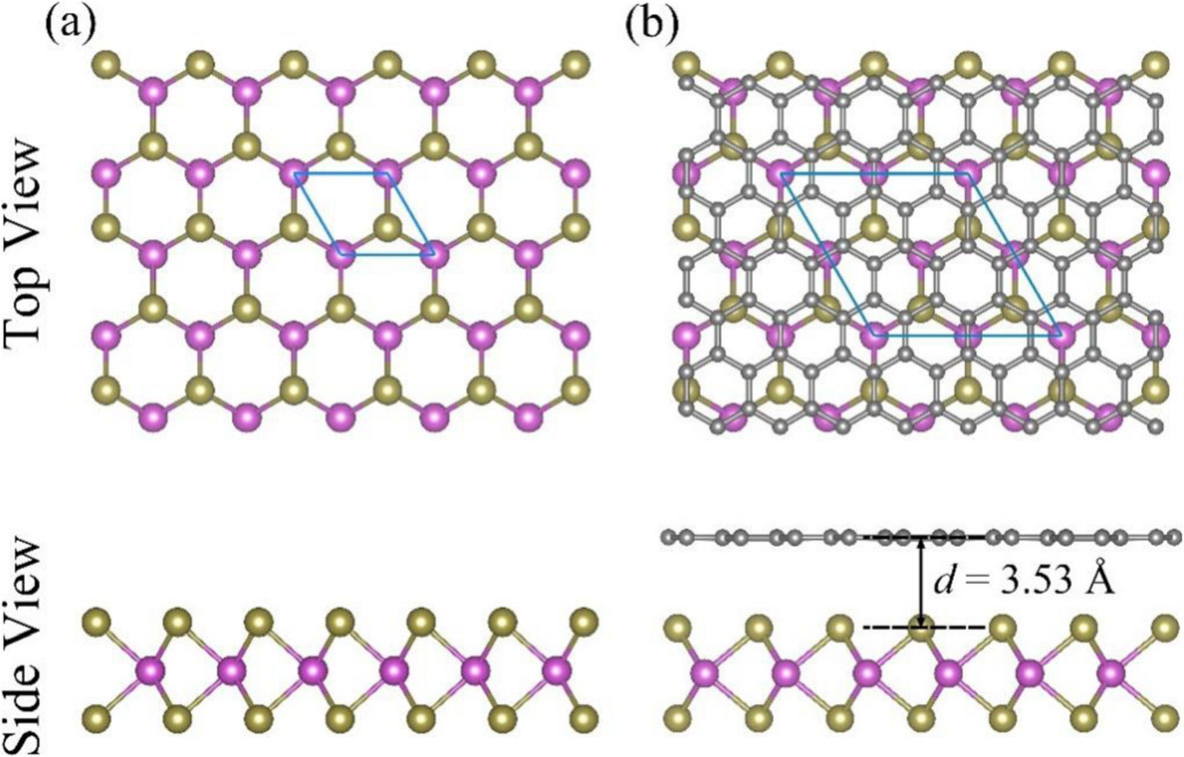
Top view and side view of the optimized structures of (**a**) MoTe_2_ monolayer and (**b**) graphene-MoTe_2_ heterostructure. The gray, pink, and green balls represent the carbon, molybdenum, and tellurium atoms, respectively. The blue parallelograms denote the 2D unit cells

**Fig. 3:**
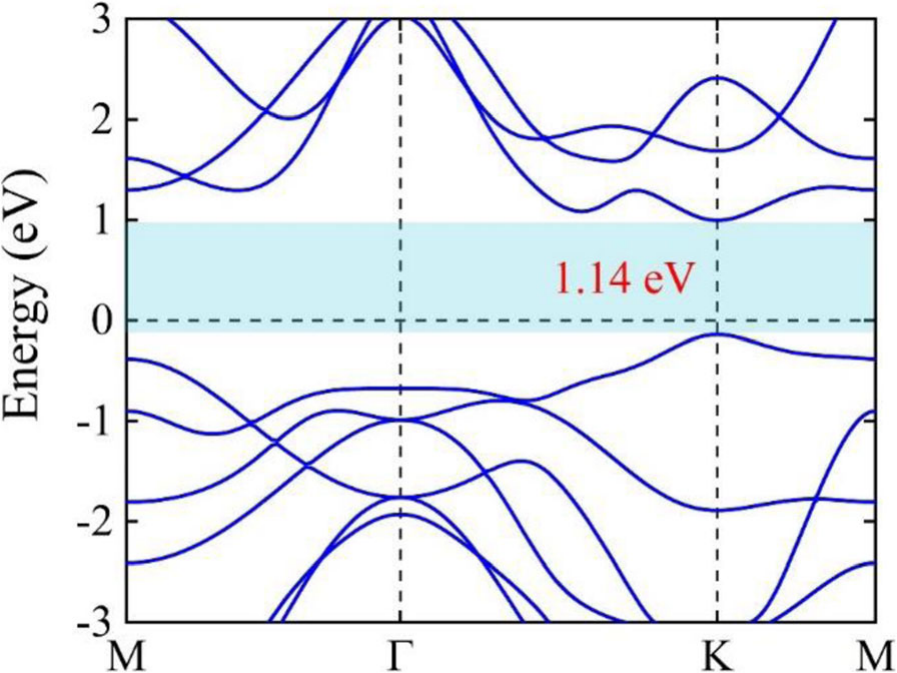
Electronic band structure of MoTe_2_ monolayer. The light blue region represents the band gap between the valence and conduction bands

The lattice crystal structures for MoTe_2_ monolayer and three typical stacking modes (HS-1, HS-2, and HS-3) of the graphene-MoTe_2_ heterostructure have all been fully optimized. The obtained binding energies of the three typical stacking modes are all nearly the same, i.e., −0.85 eV, while the equilibrium interlayer distances of the three modes are all approximately equal to 3.53 Å. Hence, we solely focus on the HS-1 graphene-MoTe_2_ heterostructure for the following discussion and omit “HS-1” for simplicity in the following text. The optimized geometry structures of MoTe_2_ monolayer and graphene-MoTe_2_ heterostructure are shown in Fig. 2. Obviously, MoTe_2_ monolayer adopts the hexagonal lattice and the optimized lattice constant is 3.52 Å, which is consistent with the experiment results [53, 54]. It can be seen clearly from the band structure of MoTe_2_ monolayer, as it is shown in Fig. 3, that MoTe_2_ monolayer is a semiconductor with a band gap of 1.14 eV, which is also consistent with the experiment results [22, 23]. When graphene and MoTe_2_ monolayer are vertically stacked up as a heterostructure, the equilibrium interlayer distance is 3.53 Å, which is comparable to the value of the Sb-MoTe_2_ heterostructure (about 3.94 Å) [55]. It could also be seen from Fig. 2 that the geometry structures of the MoTe_2_ layer and graphene layer in the graphene-MoTe_2_ heterostructure almost remain the same as the original structures of MoTe_2_ monolayer and graphene, which indicates the interaction between these two layers is weak. The binding energy of equilibrium structures −0.85 eV is lower than that of the Sb-MoTe_2_ heterostructure (about −0.37 eV) [55], so the heterostructure is energetically stable. Both the equilibrium distance between two layers and binding energy are comparable to those of typical vdW graphene-based heterostructures, such as graphene-hydrogenated phosphorus carbide [56], graphene-AsSb [29], graphene-SMoSe and graphene-SeMoS [30], and graphene-phosphorene [57], indicating that the interaction between MoTe_2_ and graphene is weak vdW type.

**Fig. 4:**
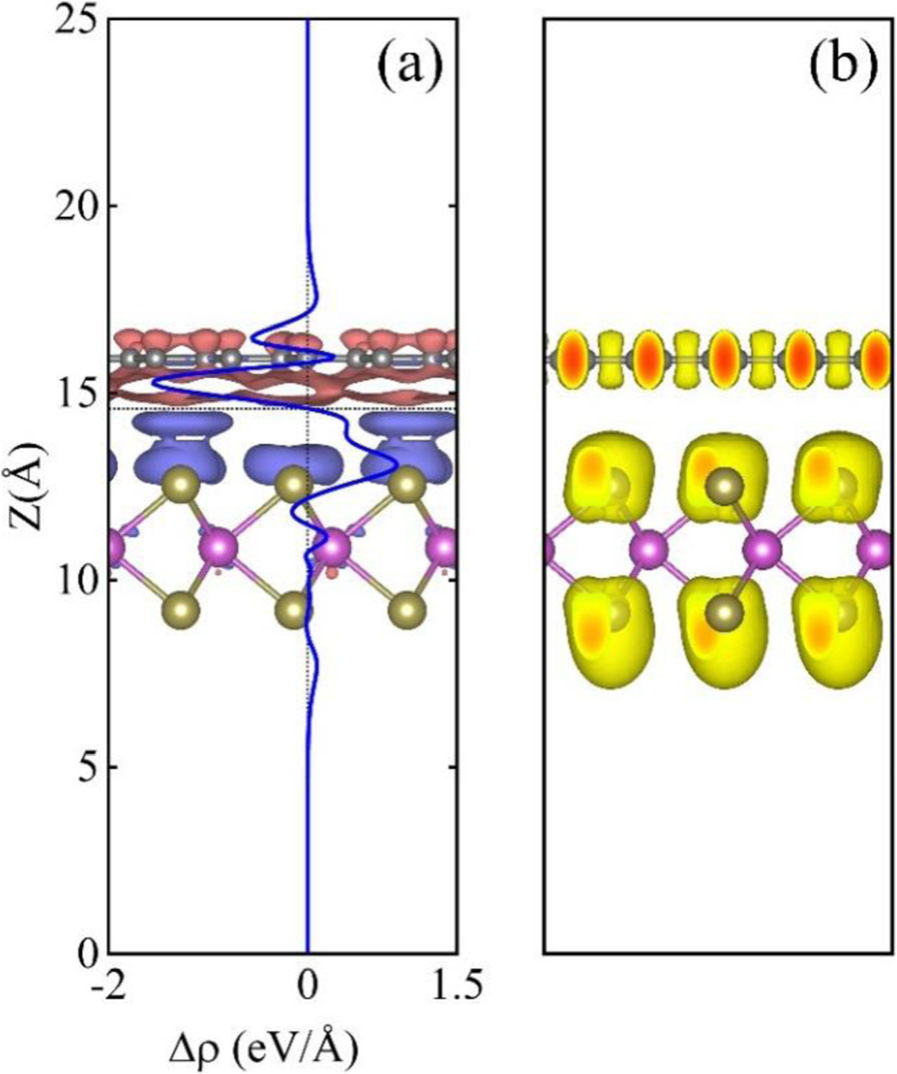
**a** The 3D charge density difference and the average charge density difference (blue line) as a function of position in the graphene-MoTe_2_ heterostructure along the *z* direction, where the blue and dark pink regions denote the accumulation and deficient of the electrons, respectively. The horizontal dashed line marks the central location between the graphene layer and MoTe_2_ layer. **b** Electron localization function of the graphene-MoTe_2_ heterostructure with the isovalue of 0.7

Actually, the charge redistribution and transfer would inevitably occur when graphene and MoTe_2_ monolayer are stacked up to form the heterostructure. The 3D charge density difference in the graphene-MoTe_2_ heterostructure defined as Δ*ρ* = *ρ*_H_ − *ρ*_G_ − *ρ*_MT_ has been calculated, where *ρ*_H_, *ρ*_G_, and *ρ*_MT_ are the charge densities of heterostructure, isolated graphene, and MoTe_2_ monolayer, respectively. The result is shown in Fig. 4a, in which the blue and dark pink regions represent charge accumulation and depletion, respectively. Obviously, the blue region is just under the MoTe_2_ layer, which indicates that the electrons accumulate around the MoTe_2_ layer; while graphene layer is surrounded by the dark pink area, implying that the holes accumulate around the graphene layer. To see the property of the charge transfer more clearly, the planar average 〈∆*ρ*〉, which is defined as the average value of the 3D charge density difference Δ*ρ* in planes with *z* = const. that are parallel to the graphene layer, is shown as a blue line in Fig. 4a, where the negative and positive values represent electron depletion and accumulation, respectively. The result verifies that some electrons transfer from graphene layer to MoTe_2_ layer, and there are oscillations in 〈*∆ρ*〉 in both the graphene and MoTe_2_ layer. The electron localization function (ELF) is also plotted in Fig. 4b, from which it can be seen that the shape of ELF around the Te atom near the graphene layer is obviously different with that around the Te atom of the other side, suggesting the existence of interlayer vdW interaction in the heterostructure.

**Fig. 5:**
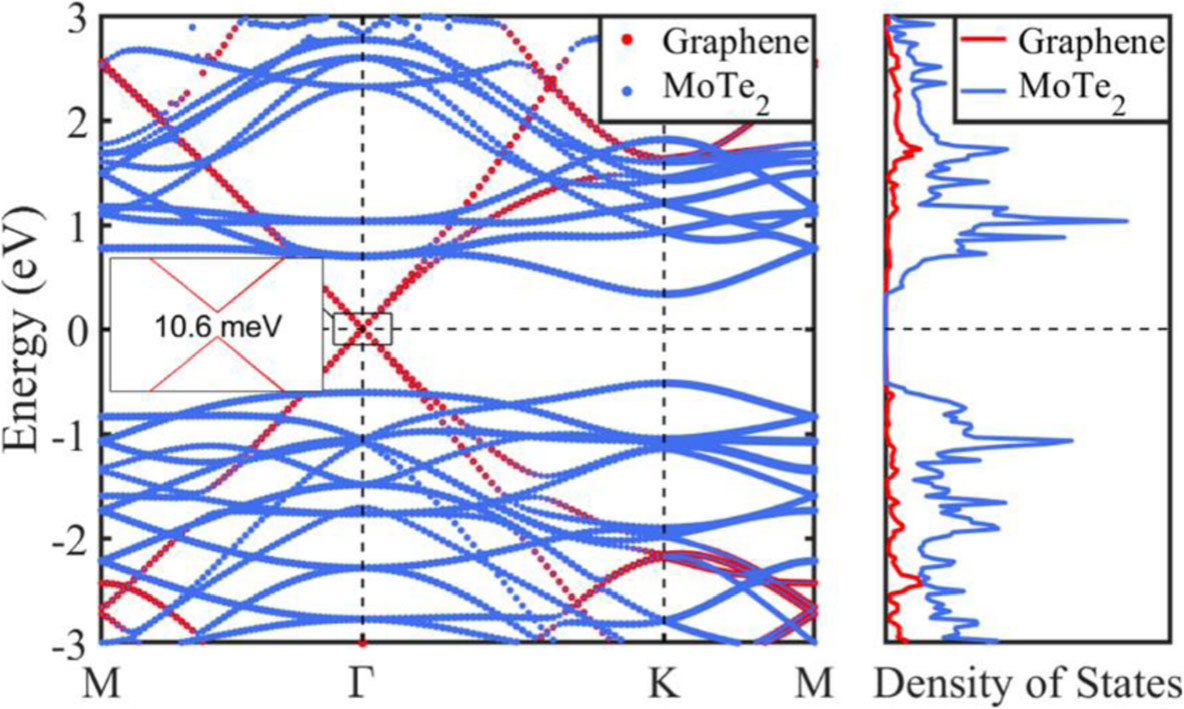
Band structures and partial density of states of graphene layer and MoTe_2_ layer in the graphene-MoTe_2_ heterostructure

Many physical properties are determined by the band structures and density of states (DOS), and the calculated band structures and DOS of the graphene-MoTe_2_ heterostructure are shown in Fig. 5, where the Fermi level is set to zero. The Dirac cone of the graphene layer around the Fermi level is still well preserved; however, a band gap of about 10.6 meV is opened up. That is to say, there is a small but noticeable interlayer coupling in the heterostructure. The bands contributed by the MoTe_2_ layer demonstrate that the semiconductor characteristics of MoTe_2_ layer with a direct band gap are retained. The band gap of MoTe_2_ layer is 0.85 eV in the heterostructure, which is changed compared with the result of 1.14 eV for the isolated MoTe_2_ monolayer. One striking feature in Fig. 5 is that the band structure can be deemed as the simple sum of the bands of isolated layers. It is not surprising that interaction between the graphene layer and the MoTe_2_ layer is insufficient to modify the characteristics of the band structure of each component in the heterostructure, so the interlayer interaction effect on the band structure is very weak. This further indicates that the vdW interaction dominates between MoTe_2_ layer and graphene layer in the heterostructure, and thus preserving the intrinsic key properties.

**Fig. 6:**
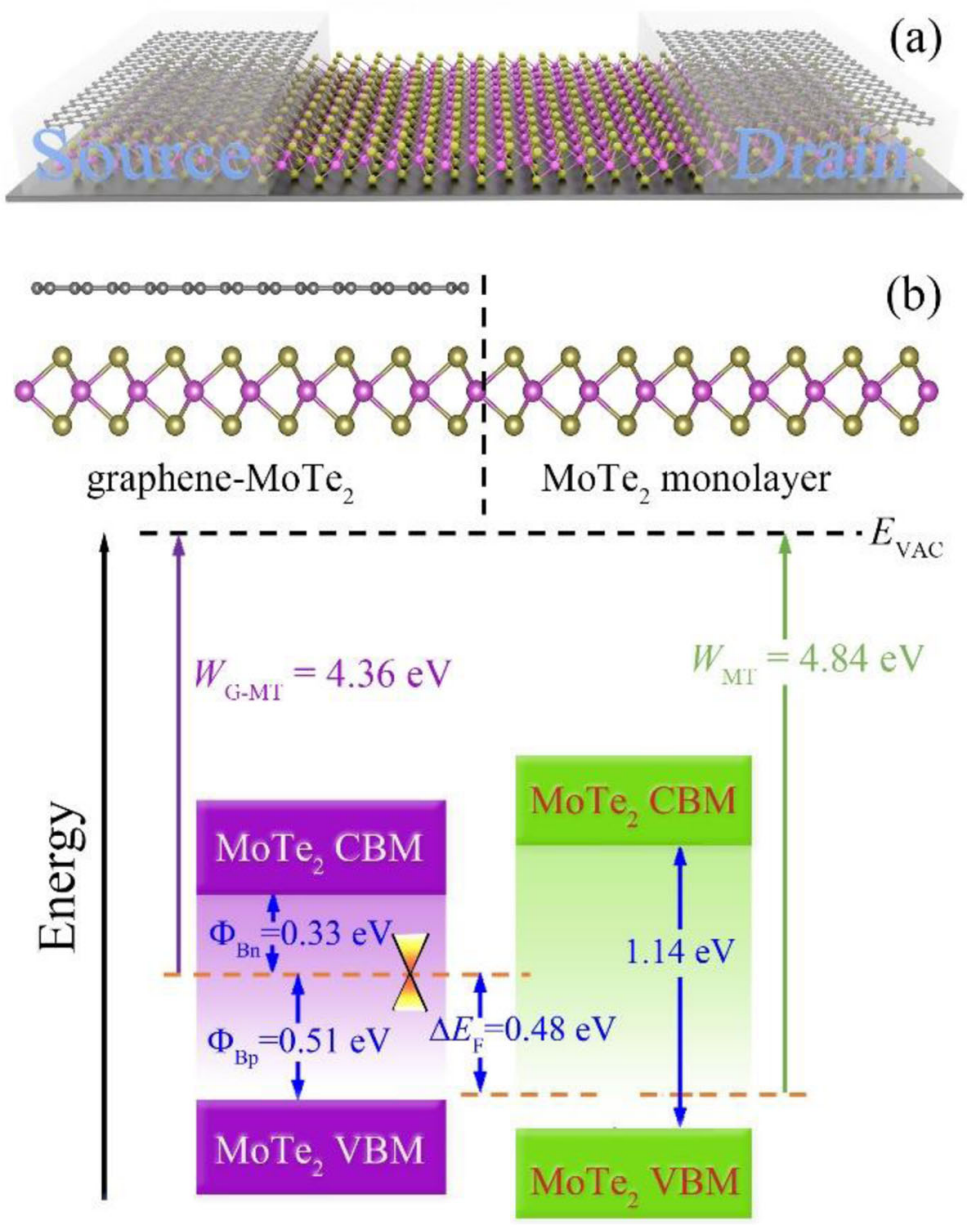
**a** The schematic diagram of a graphene-MoTe_2_ heterostructure based transistor. **b** Band alignment of graphene-MoTe_2_ heterostructure with respect to vacuum level, where the red cone represents the position of the Dirac point of graphene layer in the heterostructure. CBM and VBM represent conduction band minimum and valence band maximum, respectively. *W*_G-MT_ and *W*_MT_ are the work functions of graphene-MoTe_2_ heterostructure and MoTe_2_ monolayer, respectively

The contact properties of heterostructures are of importance for device applications. A graphene-MoTe_2_ heterojunction-based transistor has been designed, and the schematic diagram is shown in Fig. 6a, where the MoTe_2_ monolayer is used as the channel material and graphene as both source or drain and gate electrodes. Due to the difference in work functions of the metal and semiconductor, there is a band bending at the interface, which can be estimated by the Fermi level difference (Δ*E*_*F*_), defined by Δ*E*_*F*_ = *W*_G − MT_ − *W*_MT_, where *W*_G − MT_ and *W*_MT_ are the work functions of the heterostructures and the corresponding MoTe_2_ monolayer, respectively. The calculated *W*_G − MT_ and *W*_MT_ are 4.36 eV and 4.84 eV, respectively, as is shown in Fig. 6b. The results are consistent with the experimental values [39]. Consequently, the band bending (Δ*E*_*F*_) is about 0.48 eV in the heterostructure, which is comparable to the result of graphene-hydrogenated phosphorus carbide heterostructure [56].

One of the most important contact properties of metal-semiconductor heterostructures is Schottky barrier at the vertical interface (between the graphene layer and the MoTe_2_ layer), which determines the current flow across the interface of heterostructures thus playing a significant role in the corresponding device performance. In general, according to the types of the semiconductors in heterostructures, SBH is divided into *n* type and *p* type, respectively. The *n* type SBH (*Φ*_Bn_) is defined as the energy difference between the conduction band minimum (CBM) of the semiconductor (*E*_C_) and the Fermi level of the metal (*E*_F_), i.e., *Φ*_Bn_ = *E*_C_ − *E*_F_. The *p* type SBH (*Φ*_Bp_) is defined as the energy difference between the Fermi level of the metal and the valence band maximum (VBM) of the semiconductor (*E*_V_), i.e., *Φ*_Bp_ = *E*_F_ − *E*_V_. The SBH results of the graphene-MoTe_2_ heterostructure is shown in Fig. 6b. Due to the charge transfer, the Fermi level moves from the valence band side of the MoTe_2_ monolayer to conduction band side of MoTe_2_ layer in the heterostructure, which denotes that the SBH of the heterostructure is *n* type with the value of about 0.33 eV at the interface. That is to say, the charge conduction of the heterostructure will be mainly through electrons.

**Fig. 7:**
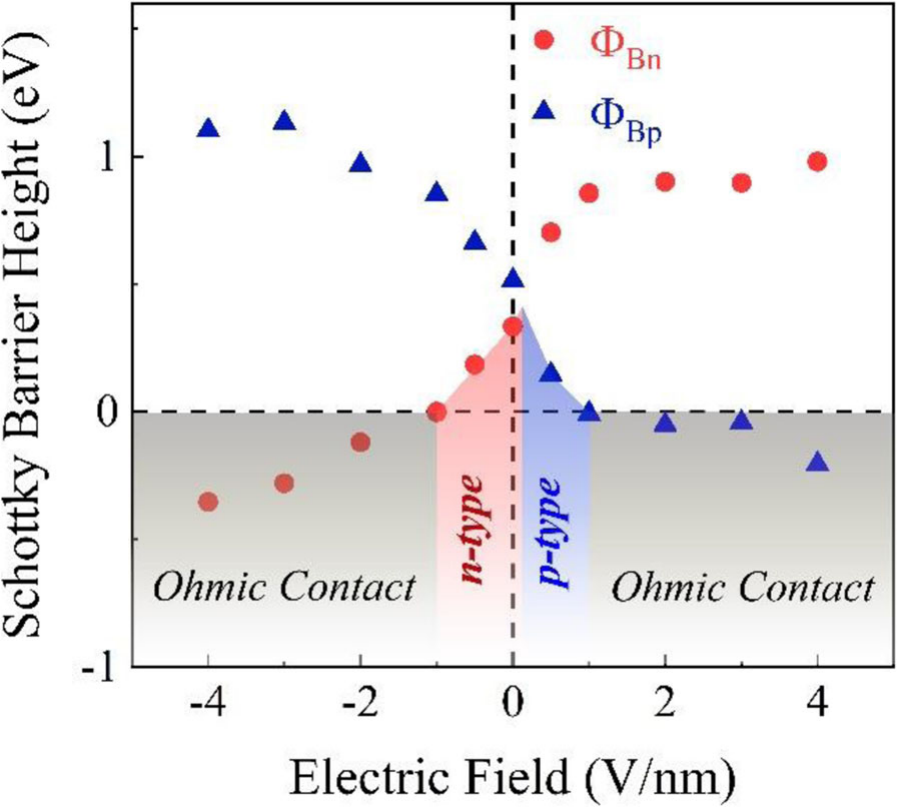
The Schottky barrier height of the graphene-MoTe_2_ heterostructure as a function of the external electric field. The blue and red areas denote the Schottky contact as *p* type and *n* type, respectively. The gray area marks the Ohmic contact region

To improve the performance of heterostructure-based transistors, it would be desirable to tune the SBH. It is demonstrated that the SBH can be tuned via applying an external electric field and in-plane strain [29, 30, 58]. A series of calculations for the band structure of the heterostructure under different external electric fields have been made, and the results are shown in Fig. 7, where the direction for the positive external electric field points from the MoTe_2_ layer to the graphene layer, while the negative value is along the opposite direction. In the Schottky contact region, *Φ*_Bn_ exhibits an approximately upward linear relationship with the electric field, while *Φ*_Bp_ behaves reversely. These results suggest that the positive and negative electric fields enable the Fermi level to shift toward the VBM and CBM of the MoTe_2_ layer in the heterostructure, respectively. Under the negative electric field, *Φ*_Bn_ is smaller than *Φ*_Bp_ all the time, indicating that the Schottky barrier is *n* type. When the positive electric field is a little greater than zero, *Φ*_Bn_ begins to be greater than *Φ*_Bp_, which means the Schottky barrier is changed from *n* type to *p* type at the graphene-MoTe_2_ interface. It is obviously that the band gap (approximately equals to the sum of *Φ*_Bn_ and *Φ*_Bp_) of the MoTe_2_ layer almost remains constant under the external electric field, which denotes that the external field has little effect on the pristine electronic properties. This can be understood as follows: although the external electric field can change the energy eigenvalues of the valence electron such as CBM and VBM, their relative values are unchanged, resulting in the band gap remaining constant. In other words, the external electric field could not change the band structure except the band bending. It can be also seen clearly from Fig. 7 that the SBH becomes negative when the positive electric field is greater than 1.0 V/nm, which means that electrons from graphene would be injected into MoTe_2_ without any barrier, indicating that MoTe_2_ possesses a metallic conductivity, and thus realizing a Schottky-to-Ohmic contact transition. For the negative electric field when the intensity exceeds 1.0 V/nm, the heterostructure could also be tuned to the Ohmic contact. All these results demonstrate that applying an external electric field is an effective strategy to modulate the SBH and contact type for the graphene-MoTe_2_ heterostructure.

**Fig. 8:**
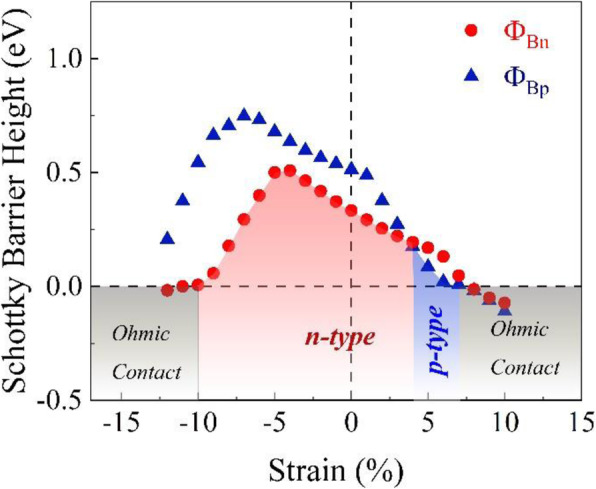
The Schottky barrier height of the graphene-MoTe_2_ heterostructure as a function of strain. The blue and red areas denote the Schottky contact as *p* type and *n* type, respectively. The gray area marks the Ohmic contact region

The SBH as a function of the in-plane biaxial strain is also calculated and the results are displayed in Fig. 8. For applying the biaxial strain, the *z* coordinate of the Te atoms are relaxed while the positions of other atoms remain fixed after changing the size of the unit cell. It is shown that strain can also tune the SBH of the heterostructure between *n* type and *p* type and drive the heterostructure to approach the Ohmic contact. The behaviors of strain dependence of SBH are very different with that of the electric field dependence. The situation becomes much more complex. For a wide strain range, *Φ*_Bn_ is smaller than *Φ*_Bp_, while only in a narrow tensile strain range *Φ*_Bp_ maintains smaller than *Φ*_Bn_. That is to say, the strain range of *n*-type SBH (the strain is about −10 ~ 4%) is much wider than that of the *p* type (about 4 ~ 7%). When the tensile strain reaches 7% and the compressive strain reaches 10%, the Ohmic contact for the heterostructure also appears. It is worth noting that the band gap of the MoTe_2_ layer in the heterostructure would change strongly with the variation of the strain in the Schottky contact region, which is strongly different with the results of the electric field case. When the lattices are under strain, they would deviate from the equilibrium state, thus causing the change of the band structure. In fact, not only the value of band gap but also the type of band gap (direct or indirect) would be change by strain. For small strain, MoTe_2_ layer remains a direct band gap while it changes to indirect band gap for large strain. Here, it should be pointed out that for a real transistor the actual conditions to realize the Schottky-to-Ohmic contact transition may be somewhat different with the calculated results due to the actual situations.

The above results suggest that both applying an external electric field and in-plane biaxial strain are effective methods to control SBH and the type of contact of the graphene-MoTe_2_ heterostructure, which is indispensable to design 2D vdW heterostructure based field-effect transistors. Furthermore, the graphene-MoTe_2_ heterostructure can be applied for tunable Schottky diodes in nanoelectronic and optoelectronic devices.

## Conclusions

In summary, the band structures of the graphene-MoTe_2_ heterostructure under different electric fields and biaxial strains have been systematically investigated based on first-principle calculations. The electronic structures of graphene and MoTe_2_ are well preserved after being stacked up together along the vertical direction, which suggests that the interlayer interaction of the heterostructure belongs to the vdW type. However, the Fermi level moves toward CBM of the MoTe_2_ layer after the formation of the heterostructure, i.e., the Schottky contacts are *n* type with a 0.33 eV SBH. The SBH and the type of contacts at the heterostructure interface can be effectively modulated by applying an external electric field or strain. When an electric field is applied, in the Schottky contact region, the *n* type SBH exhibits an approximately upward linear relationship with the electric field, and *p* type SBH behaves reversely. The heterostructure can be tuned to the Ohmic contact for an electric field which is greater than 1.0 V/nm at both positive and negative sides. For the case of applying biaxial strain, the situation is more complex than the electric field case. The strain range of *n* type SBH is much wider than that of the *p* type. When the tensile strain reaches 7% or the compressive strain reaches 10%, the Ohmic contact also appears. All the results demonstrate that applying an electric field or strain is a good way to control of the SBH as well as the type of contact of the heterostructure, even drive the system into the Ohmic contact. These features are quite significant for designing high performance nanoelectronic and optoelectronic devices.

## Data Availability

The datasets supporting the conclusions of this article are included within the article, and further information about the data and materials could be made available to the interested party under a motivated request addressed to the corresponding author.

## Abbreviations

2D: Two-dimensional
TMDs: Transition metal dichalcogenides
vdW: Van der Waals
SBH: Schottky barrier height
DFT: Density functional theory
PAW: Projector augmented wave
PBE: Perdew-Burke-Ernzerhof
GGA: Generalized gradient approximation
DOS: Density of states
CBM: Conduction band minimum
VBM: Valence band maximum
